# Global Burden of Double Malnutrition: Has Anyone Seen It?

**DOI:** 10.1371/journal.pone.0025120

**Published:** 2011-09-28

**Authors:** Daniel J. Corsi, Jocelyn E. Finlay, S. V. Subramanian

**Affiliations:** 1 Population Health Research Institute, McMaster University and Hamilton Health Sciences, Hamilton, Canada; 2 Department of Global Health and Population, Harvard School of Public Health, Boston, Massachusetts, United States of America; 3 Department of Society, Human Development and Health, Harvard School of Public Health, Boston, Massachusetts, United States of America; Indiana University, United States of America

## Abstract

**Background:**

Low- to middle-income countries (LMICs) are believed to be characterized by the coexistence of underweight and overweight. It has also been posited that such coexistence is appearing among the low socioeconomic status (SES) groups.

**Methods:**

We conducted a cross-sectional analysis of nationally representative samples of 451321 women aged 20–49 years drawn from 57 Demographic and Health Surveys conducted between 1994 and 2008. Body Mass Index (BMI in kg/m^2^), was used to define underweight and overweight following conventional cut-points. Covariates included age, household wealth, education, and residence. We estimated multinomial multilevel models to assess the extent to which underweight (BMI<18.5 kg/m^2^) and overweight (BMI≥25.0 kg/m^2^) correlate at the country-level, and at the neighborhood-level within each country.

**Results:**

In age-adjusted models, there was a strong negative correlation between likelihood of being underweight and overweight at country- (*r* = −0.79, *p*<0.001), and at the neighborhood-level within countries (*r* = −0.51, *P*<0.001). Negative correlations ranging from −0.11 to −0.90 were observed in 46 of the 57 countries at the neighborhood-level and 29/57 were statistically significant (*p*≤0.05). Similar negative correlations were observed in analyses restricted to low SES groups. Finally, the negative correlations across countries, and within-countries, appeared to be stable over time in a sub-set of 36 countries.

**Conclusion:**

The explicitly negative correlations between prevalence of underweight and overweight at the country-level and at neighborhood-level suggest that the hypothesized coexistence of underweight and overweight has not yet occurred in a substantial manner in a majority of LMICs.

## Introduction

It is commonly asserted that low- to middle-income countries (LMICs) are characterized by the coexistence of underweight and overweight [1]–[4], referred to as the “double burden of malnutrition”. Furthermore, studies in LMICs have suggested that burdens of overweight and obesity has or will shift to lower socioeconomic status (SES) groups as countries develop economically, exposing these groups simultaneously to under- and overnutiriton [5]–[6]. Therefore, it is imperative that the patterning of underweight and overweight be examined in transitioning LMICs to inform health policies for the management of the nutrition-related burden of disease [7]–[8]. Although the double burden of malnutrition is plausible within the context of epidemiologic transition away from a predominance of nutritional deficiencies towards a greater burden of noncommunicable disease [8]–[9], the majority of studies that have thus far reported the coexistence of underweight and overweight within populations have been based on the prevalence of these conditions, making their interpretation problematic without an appropriate reference by which to determine the occurrence of a double burden [5], [10]–[19]. An overview of studies among men and women in transitional countries from Latin America, Asia, and Africa that have reported the coexistence of underweight and overweight using prevalence is presented in **Table 1**. A determination of the coexistence of underweight and overweight in a population can alternatively be made through a formal, multilevel examination of the residual covariance as a measure of the correlation in the prevalence of underweight and overweight at a given geographic level [20]. A strong negative correlation, at a given geographic scale, would suggest the absence of coexistence of underweight and overweight, while a strong positive correlation would suggest the simultaneous presence of the twin burden of nutrition. To our knowledge, we are not aware of any formal empirical assessment of the coexistence of underweight and overweight that is comparable between countries and small areas within countries. Using the largest available, comparable, and most recent sample of adult women in the reproductive age group from 57 LMICs with objective measurements of height and weight, we assessed the extent to which underweight and overweight coexist in the general population and among low SES groups, at both the country-level and at the level of neighborhoods within countries. In a sub-set of countries, we also assessed whether the observed patterns of coexistence have changed over time.

**Table 1:** Studies reporting a double burden of nutrition among adults in low- to middle-income countries.

				Body Mass Index (BMI, kg/m^2^)		% Underweight		% Overweight/Obese	
Country	Year	Sample (*n*)	Age group (years)	Underweight Criterion	Overweight/obese Criterion	Males	Females	Males	Females
Latin America									
Brazil [10]	1989	7160	20–64	<18.5	≥30.0	-	6.6	4.6	12.3
Brazil	1997	5137	20–64	<18.5	≥30.0	-	6.0	6.9	12.7
Mexico [11]	1994	2125	20–69	<18.5	25–29.9	1.9	1.5	36.1	35.8
Mexico	2000	45249	20–75	<18.5	25–29.9	1.8	1.7	40.5	36.1
Asia									
Bangladesh [12]	2004	242433 (Rural)	15–45	<18.5	≥25.0	-	38.8	-	4.1
Bangladesh	2004	39749 (Urban poor)	15–45	<18.5	≥25.0	-	29.7	-	9.1
China [13]	1989	4527	20–45	<18.5	≥25.0	8.4	8.9	6.5	11.4
China	2000	4046	20–45	<18.5	≥25.0	6.3	6.7	20.2	19.3
India [14]	1994	99598 (Urban)	35+	<18.5	≥25.0	19.5	19.1	19.2	29.7
India [15]	2001	4032	15–49	<18.5	≥25.0	-	37.0	-	12.0
Indonesia [16] *	2007	-	≥15	<18.5	≥25.0	15.0	19.0		
Vietnam [17]	2000	14452	25–64	<18.5	≥25.0	22.0	27.9	2.8	5.5
Vietnam	2005	17213	25–64	<18.5	≥25.0	19.9	21.9	5.3	8.0
Africa									
Egypt [11]	2004	19201	≥20	17.0–18.5	25.0–29.9	2.8	2.0	35.4	26.9
Gambia [18] *	1996–1997	5373	≥15	<18.0	≥30.0	18.0	2.3		
South Africa [19]	1990	976 (Cape Peninsula)	15–64	<20.0 (male), <19.0 (female)	≥25.0 (male), ≥24.0 (female)	26.3	9.8	17.7	35.2
LMICs (Urban) [6]	1997	157844 (36 countries)	20–49	<18.5	≥25.0	-	5.9	-	32.4
LMICs (Rural)	-	-	-	-	-	-	9.3	-	19.4

Notes:

*Male and Female populations combined.

## Methods

### Data

Data were obtained from single and repeated cross-sectional Demographic and Health Surveys (DHS) conducted between 1992 and 2008 in 57 LMICs (**Table 2**) [21]. The MEASURE DHS project has provided assistance in conducting standardized household sample surveys in LMICs since 1984 with a focus on health, socioeconomic, nutrition, and fertility-related information from all women in the reproductive age group (15–49 years) [22]. In some DHS surveys, the population was limited to ever-married women of reproductive age with children. Strengths of the DHS data for studying population health include very high response rates, national coverage, standard data collection procedures, and interviewer training [23]–[25]. The core DHS questionnaire has been standardized and pretested to ensure comparability across populations and over time. The DHS program has developed a rigorous area-based sampling design which employs multistage stratification and probabilistic sampling with each areal unit having a defined probability of selection [26]. In each included survey, sampling was stratified according to urban and rural areas and in some countries by additional geographic or administrative regions. The general DHS sampling framework was adapted to produce country-specific sample designs [26]. **Table 2** describes each survey by country and year, sample sizes, number of neighborhoods, and the percentage prevalence of underweight and overweight for all women aged 20 to 49 years. For 36 countries with repeated surveys, 2 rows are provided indicating the first and most recent survey conducted in that country. **Table S1** describes the sampling plans for each survey by country and year, including response rates and anthropometric (height and weight) measurement protocol.

**Table 2:** Survey year, sample size, number of neighborhoods, and distribution of nutritional status for women aged 20–49 in 57 low- to middle-income countries.

Country	Survey Year	Sample Size		Nutritional status according to body mass index (BMI, kg/m^2^) classification					
			Neighborhoods	Underweight (<18.5)		Normal BMI (18.5–24.9)		Overweight (≥25)	
		*n*	*n*	*N*	*%*	*n*	*%*	*n*	*%*
Total (latest survey)	2005 (median)	451321	32814	53713	11.9	254549	56.4	143059	31.7
Albania	2008	5898	450	90	1.5	3143	53.3	2665	45.2
Armenia	2005	5058	308	178	3.5	2351	46.5	2529	50.0
	2000	4891	259	131	2.7	2443	49.9	2317	47.4
Azerbaijan	2006	6461	318	212	3.3	2794	43.2	3455	53.5
Bangladesh	2007	9028	361	2504	27.7	5169	57.3	1355	15.0
	1996	3375	313	1729	51.2	1535	45.5	111	3.3
Benin	2006	12280	750	1034	8.4	8757	71.3	2489	20.3
	1996	2136	200	314	14.7	1627	76.2	195	9.1
Bolivia	2003	12300	999	140	1.1	5222	42.5	6938	56.4
	1994	2127	555	46	2.2	1345	63.2	736	34.6
Brazil	1996	2883	759	174	6.0	1702	59.0	1007	34.9
Burkina Faso	2003	8478	400	1663	19.6	5986	70.6	829	9.8
	1992	3189	230	426	13.4	2430	76.2	333	10.4
Cambodia	2005	6147	557	1093	17.8	4357	70.9	697	11.3
	2000	5292	471	1011	19.1	3897	73.6	384	7.3
Cameroon	2005	3467	461	181	5.2	2154	62.1	1132	32.7
	1998	1426	200	95	6.7	972	68.2	359	25.2
Central African Republic (CAR)	1995	1760	229	272	15.5	1367	77.7	121	6.9
Chad	2004	2618	196	541	20.7	1782	68.1	295	11.3
	1996	3261	246	684	21.0	2332	71.5	245	7.5
Colombia	2005	27654	3792	1014	3.7	13353	48.3	13287	48.0
	1995	3065	897	102	3.3	1683	54.9	1280	41.8
Comoros	1996	743	98	72	9.7	519	69.9	152	20.5
Congo, Dem. Rep.	2007	3308	300	502	15.2	2337	70.6	469	14.2
Congo, Rep.	2005	4874	225	550	11.3	2906	59.6	1418	29.1
Cote d'Ivoire	1998–9	2005	140	136	6.8	1316	65.6	553	27.6
	1994	2682	246	226	8.4	2049	76.4	407	15.2
Dominican Republic	1996	5820	395	366	6.3	2906	49.9	2548	43.8
Egypt	2008	14411	1263	89	0.6	3223	22.4	11099	77.0
	1995	6497	921	124	1.9	3125	48.1	3248	50.0
Ethiopia	2005	4644	534	1144	24.6	3165	68.2	335	7.2
	2000	10523	539	3031	28.8	6850	65.1	642	6.1
Gabon	2000	2082	247	141	6.8	1373	65.9	568	27.3
Ghana	2008	3490	411	243	7.0	2112	60.5	1135	32.5
	1993	1650	388	193	11.7	1236	74.9	221	13.4
Guatemala	1998–9	4547	405	149	3.3	2928	64.4	1470	32.3
Guinea	2005	2834	295	342	12.1	2061	72.7	431	15.2
	1999	2983	292	345	11.6	2239	75.1	399	13.4
Haiti	2005–6	3632	339	453	12.5	2260	62.2	919	25.3
	1994	1788	171	327	18.3	1241	69.4	220	12.3
Honduras	2005	13988	1046	366	2.6	6555	46.9	7067	50.5
India	2005	91243	3849	24281	26.6	50367	55.2	16595	18.2
	1998	72469	3473	22860	31.5	40182	55.4	9427	13.0
Jordan	2007	4446	464	55	1.2	1265	28.5	3126	70.3
	1997	3000	300	69	2.3	1070	35.7	1861	62.0
Kazakhstan	1999	1880	205	117	6.2	1086	57.8	677	36.0
	1995	2900	176	180	6.2	1486	51.2	1234	42.6
Kenya	2003	6046	398	656	10.9	3605	59.6	1785	29.5
	1998	3009	526	333	11.1	2205	73.3	471	15.7
Kyrgyz Republic	1997	2871	162	151	5.3	1840	64.1	880	30.7
Lesotho	2004	2401	403	103	4.3	1172	48.8	1126	46.9
Liberia	2007	4991	298	405	8.1	3373	67.6	1213	24.3
Madagascar	2003–4	5909	594	1453	24.6	3961	67.0	495	8.4
	1997	2253	265	431	19.1	1710	75.9	112	5.0
Malawi	2004	7746	521	618	8.0	5969	77.1	1159	15.0
	1992	2101	225	169	8.0	1684	80.2	248	11.8
Mali	2006	9774	406	1018	10.4	6631	67.8	2125	21.7
	1995	3787	300	602	15.9	2823	74.5	362	9.6
Moldova	2005	5709	400	199	3.5	2715	47.6	2795	49.0
Morocco	2003–4	12713	480	668	5.3	6681	52.6	5364	42.2
	1992	2795	211	109	3.9	1758	62.9	928	33.2
Mozambique	2003	8327	604	615	7.4	6260	75.2	1452	17.4
	1997	2823	392	251	8.9	2253	79.8	319	11.3
Namibia	2006–7	6916	500	835	12.1	3733	54.0	2348	34.0
	1992	2061	162	276	13.4	1364	66.2	421	20.4
Nepal	2006	7833	260	1808	23.1	5277	67.4	748	9.5
	1996	3068	253	767	25.0	2238	72.9	63	2.1
Nicaragua	2001	9098	610	230	2.5	3990	43.9	4878	53.6
	1997	9290	592	284	3.1	4600	49.5	4406	47.4
Niger	2006	3126	342	445	14.2	2005	64.1	676	21.6
	1998	2958	267	556	18.8	2075	70.1	327	11.1
Nigeria	2008	23063	886	2519	10.9	14981	65.0	5563	24.1
	2003	5029	362	598	11.9	3258	64.8	1173	23.3
Peru	2004	20943	1285	237	1.1	9234	44.1	11472	54.8
	1991	4886	876	61	1.2	2917	59.7	1908	39.1
Rwanda	2005	3911	462	295	7.5	3119	79.7	497	12.7
	2000	6628	441	451	6.8	5159	77.8	1018	15.4
Senegal	2005	3059	376	393	12.8	1893	61.9	773	25.3
Sierra Leone	2008	2692	353	273	10.1	1545	57.4	874	32.5
South Africa	1998	4263	935	241	5.7	1652	38.8	2370	55.6
Swaziland	2006	3412	274	60	1.8	1331	39.0	2021	59.2
Tanzania	2004–5	7064	475	698	9.9	4928	69.8	1438	20.4
	1996	3502	354	331	9.5	2689	76.8	482	13.8
Togo	1998	3113	282	343	11.0	2435	78.2	335	10.8
Turkey	1998	2210	460	52	2.4	949	42.9	1209	54.7
	1993	2294	463	49	2.1	1046	45.6	1199	52.3
Uganda	2006	1925	367	221	11.5	1376	71.5	328	17.0
	1995	2827	293	254	9.0	2246	79.4	327	11.6
Uzbekistan	1996	3182	168	233	7.3	2039	64.1	910	28.6
Zambia	2007	4846	319	391	8.1	3393	70.0	1062	21.9
	1996	3483	312	328	9.4	2715	78.0	440	12.6
Zimbabwe	2005–6	6199	398	451	7.3	3946	63.7	1802	29.1
	1994	1774	230	95	5.4	1276	71.9	403	22.7

Notes: Percent underweight represents the number of women with a BMI of less than 18.5 divided by the total number of women and then multiplied by 100; percent normal weight represent percent overweight represents the number of women with a BMI between 18.5 and 24.9 divided by the total number of and then multiplied by 100; percent overweight represents the number of women with a BMI of 25 and above divided by the total number of women and then multiplied by 100. Percentages are calculated as row percentages.

### Study population and sample size

The study population included all non-pregnant women between the ages of 20–49 years either with or without children of any age (*n* = 567047) and was derived from the most recent survey in all countries participating in the DHS anthropometric measurement module. There were 109981 (19.4% of the sample) women for whom height and weight was intentionally not measured (see **Table S1** for anthropometric measurement protocol by survey). Among those for whom height or weight should have been measured, 5682 women (1.2%) did not have a height or weight measure in the data, and 34 women (<1%) had a biologically implausible height (less than 100 cm or greater than 200 cm) or weight (less than 25 kg or greater than 200 kg) and were excluded. Twenty nine observations (<1%) were missing data on covariates.

The final sample for the primary analysis was 451321 adult women surveyed and measured between 1994 and 2008 in 57 countries. For the secondary analysis on a subset of 36 countries, a sample of 197822 women was available from the first survey conducted in these countries and a sample of 363264 women was available from the most recent survey conducted.

### Outcome

Trained field investigators weighed each woman using a solar-powered electronic scale with a precision of ±100 g, and height was measured for each woman using an adjustable measuring board accurate to 1 millimeter [27]. Body Mass Index (BMI), calculated as weight in kilograms divided by the square of height in meters (kg/m^2^) was used to classify women as underweight (BMI<18.5), normal (BMI 18.5–24.5), and overweight (BMI≥25.0) according to WHO recommendations [28].

### Independent Variables

The following covariates were considered in our analyses: age, education, household wealth (as a measure of SES), and place of residence (urban or rural). Age (20–49 y) was specified in 5-y groups. Women's education level was categorized as: no education, primary education, or having completed secondary or higher level education. Women's SES was based on a household wealth index derived from dwelling characteristics (e.g. source of drinking water) and ownership of material possessions (e.g., television, bicycle) with each woman assigned a wealth score based on the weighted combination of characteristics and assets in their household with the weights derived according to a principle component analysis (PCA) procedure [29]. We used the survey-specific household wealth index that was provided by the DHS. For each survey, PCA was conducted on the set of indicator variables representing each household characteristic or asset. Then, for each household, the values of the indicator variables were multiplied by the coefficient from the first principle component, summed, and standardized to produce the household wealth index value with a mean of 0 and a standard deviation of 1. For each survey, the sample was divided into fifths from richest to poorest along the resulting standardized index [30]–[32]. Place of residence indicated whether the household was located in an urban or rural area by census definition.

### Defining Small Areas (“Neighborhood”) within Countries

DHS surveys make use of area-based sampling and cover the entire geographical territory of each country. The cluster is the smallest unit used in the DHS area sampling frames. Sampling frames were obtained from existing country master samples, or lists of enumeration areas from a recently completed census [26]. Selected clusters to be included in the survey were checked for completeness and lists of dwellings, households, and individuals were created by field teams in each country. Larger clusters were further segmented into the DHS standard size of about 500 individuals or 100–150 households during fieldwork. For the present study, clusters, typically villages or groups of villages in rural areas and municipal wards or divisions in urban areas were taken to represent a women's residential context. In addition to being of similar size, clusters were defined using meaningful geographic characteristics and natural boarders such as rivers or mountains or other identifiable boundaries such as roads, railways, or eclectic power lines [26]. Clusters in many countries follow administrative boundaries which provide practical relevance for defining residential context.

### Analysis

Given the multilevel structure of the data and with an explicit interest in modeling the multiple categories of BMI and their correlation at the geographic level of countries and neighborhoods within countries, a multilevel multinomial modeling approach was adopted [20], [33]–[34]. Formally, 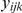 was the categorical outcome with 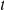 categories, for woman 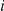 in neighborhood 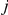 and country 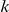. We denote the probability of being in BMI category 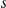 by 
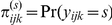
. In a multinomial logistic model, one of the outcome categories is taken as the reference categories, just as the category coded ‘0’ is normally taken as the ‘reference’ category in more the commonly used binary response models. Using the normal weight category of BMI (18.5–24.9 kg/m^2^) as the reference, we estimated a set of 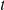
−1 logistic regressions for the underweight and overweight categories, contrasting each of the categories with the reference category as: 
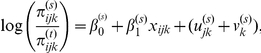
 where 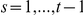. Separate intercept and slope parameters were estimated for the underweight and overweight categories, indicated by the (*s*) superscripts. The notation 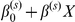 represents the fixed part of the model, and 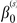, the intercept, gives the overall log odds of being underweight (or overweight) for an individual in the reference category and 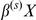 is the effect of a 1-unit increase in 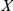 (the set of predictor variables) on the log odds of being underweight (or overweight) contrasted with the reference category (normal weight). The terms 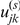, and 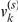 represent the random effects associated with neighborhoods and countries, assumed to be Normally distributed with mean 0 and variances, 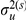 and 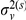. The random effects are specific to each of the contrasted category, as indicated by the 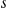 superscript, because different unobserved factors, at each level, may affect each contrast. The covariance in the random effects for underweight (*s*) and overweight (*r*) can be estimated at each level, for example, 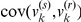 for the country level, and is the key parameter of interest for our study. For ease of interpretation, covarainces are presented in terms of correlation coefficients (*r*), and vary between −1 and 1.

We additionally estimated country-specific 2-level models (women within neighborhoods) for each country and repeated all models separately for urban and rural samples. To assess the correlation in underweight and overweight among women of low socioeconomic status (SES), we also estimated global and country-specific models restricted to the poorest quarter of women, based on household wealth. Finally, we conducted sensitivity analyses by repeating the global and country-specific models on samples of men (aged 15–54 y) from seven countries in this study (Azerbaijan, Egypt, India, Swaziland, Uganda, Colombia, and South Africa) where anthropometric measurements were available for adult men in surveyed households. In all analyses, regression and variance parameters were estimated using Markov chain Monte Carlo (MCMC) simulation and the Metropolis-Hastings algorithm, available in the statistical software *MLwiN* (version 2.20) [33], [35]–[36].

### Ethical Review

The DHS data collection procedures were approved by the ORC Macro (Calverton, Maryland) Institutional Review Board as well as by the relevant body in each country which approves research studies on human subjects. Oral informed consent for the interview/survey was obtained from respondents by interviewers. The study was reviewed by Harvard School of Public Health Institutional Review Board and was considered exempt from full review because the study was based on an anonymous public use data set with no identifiable information on the survey participants.

## Results

**Figure 1:**
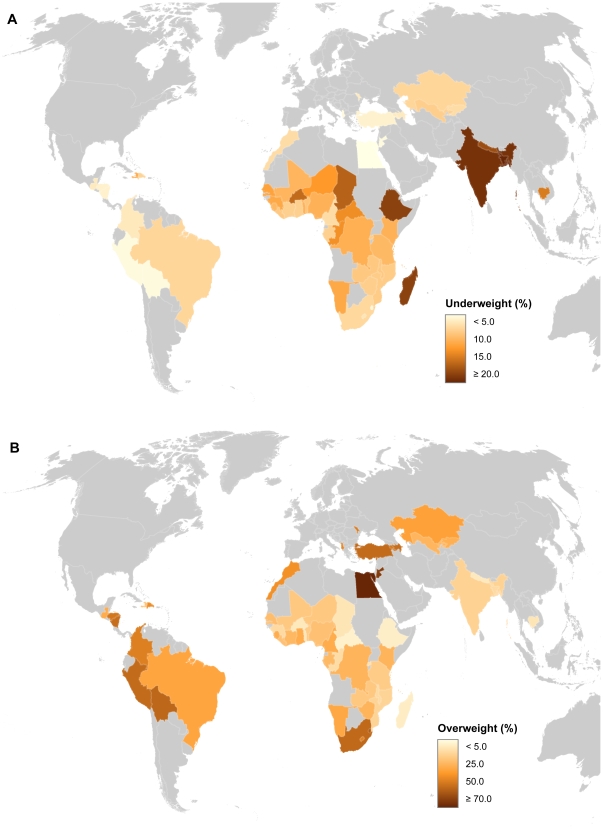
Prevalence of underweight (BMI<18.5 kg/m^2^) (A) and overweight (BMI≥25.0 kg/m^2^) (B) among women aged 20–49 in 57 low- to middle-income countries.

In the pooled sample, the prevalence of underweight and overweight was 11.9% and 31.7%, respectively in the most recent survey (**Table 1**). Within countries, underweight ranged from <1% (Egypt) to 27.7% (Bangladesh) and overweight ranged from 6.9% (Central African Republic) to 77.0% (Egypt). **Figure 1** displays the patterning of underweight and overweight across the 57 countries studied. Several countries including Chad, India, Namibia, Senegal, Niger, and Sierra Leone appeared to have a sizeable prevalence of underweight and overweight in their populations (Table 1). Among the 36 countries where repeated surveys were available, the prevalence of overweight increased in 92% at an average rate of 6.9% per year over median period of 10 years. Among the same countries the levels of underweight decreased in 64% (23/36) but at a much slower rate (<1% per year) over the same interval.

**Figure 2:**
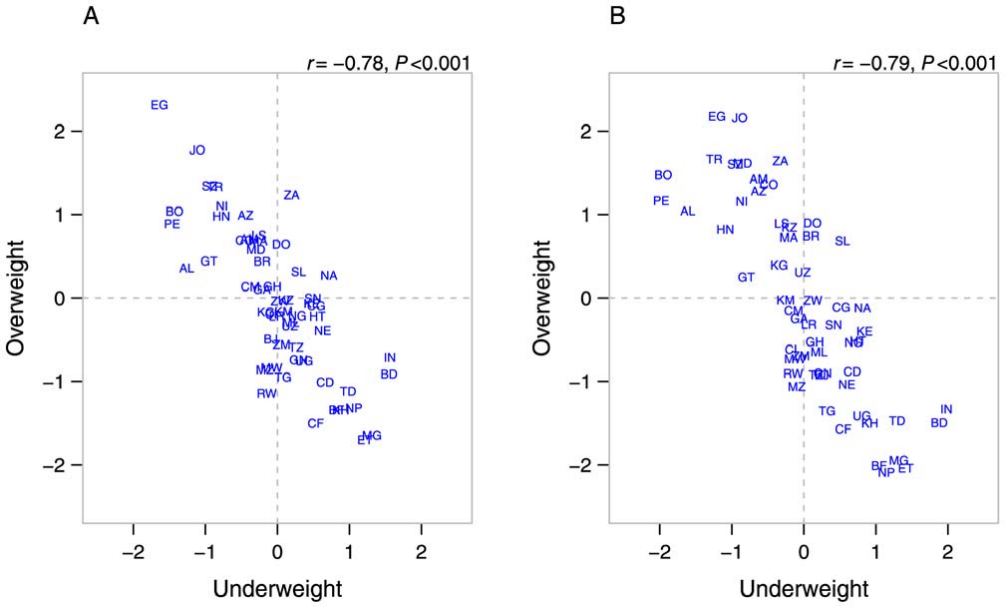
Scatter plots of country-level residuals for underweight and overweight among women aged 20–49 in 57 low- to middle-income countries overall (A), and in low-socioeconomic status groups (B). Notes: Panel A adjusted for age, education, household wealth, and place of residence, Panel B adjusted for age and urban/rural place of residence. **Country abbreviations**: AL Albania, AM Armenia, AZ Azerbaijan, BD Bangladesh, BJ Benin, BO Bolivia, BR Brazil, BF Burkina Faso, KH Cambodia, CM Cameroon, CF Central African Republic, TD Chad, CO Colombia, KM Comoros, CD Congo, Dem. Rep., CG Congo, Rep., CI Cote d'Ivoire, DO Dominican Republic, EG Egypt, ET Ethiopia, GA Gabon, GH Ghana, GT Guatemala, GN Guinea, HT Haiti, HN Honduras, IN India, JO Jordan, KZ Kazakhstan, KE Kenya, KG Kyrgyz Republic, LS Lesotho, LR Liberia, MG Madagascar, MW Malawi, ML Mali, MD Moldova, MA Morocco, MZ Mozambique, NA Namibia, NP Nepal, NI Nicaragua, NE Niger, NG Nigeria, PE Peru, RW Rwanda, SN Senegal, SL Sierra Leone, SZ Swaziland, TZ Tanzania, TG Togo, TR Turkey, UG Uganda, UZ Uzbekistan, ZM Zambia, ZW Zimbabwe, ZA South Africa.

Globally, the country-level correlation between the underweight and overweight was −0.79 (*P*<0.001) in the age-adjusted model. Further covariate adjustment did not alter the country-level correlation (**Figure 2A**, underweight/overweight *r* = −0.78, *P*<0.001). These patterns were largely repeated among low SES groups (**Figure 2B**). In these figures, a zero on each axis represents the global average for underweight and overweight. Countries estimated to have above average prevalence of both underweight and overweight would appear in the upper right quadrant of these plots. South Africa, Sierra Leone, Namibia, and additionally Brazil and Dominican Republic in the low SES model, were found to simultaneously have above average underweight and overweight at the national level.

In the pooled age-adjusted model that also accounted for between-country differences, neighborhoods with higher levels of underweight (overweight) were more likely to have lower levels of overweight (underweight) (*r* = −0.51, *P*<0.001). Additional adjustment for socioeconomic status and place of residence covariates reduced the magnitude of this correlation, but it remained statistically significant (*r* = −0.31, *P*<0.001).

**Figure 3:**
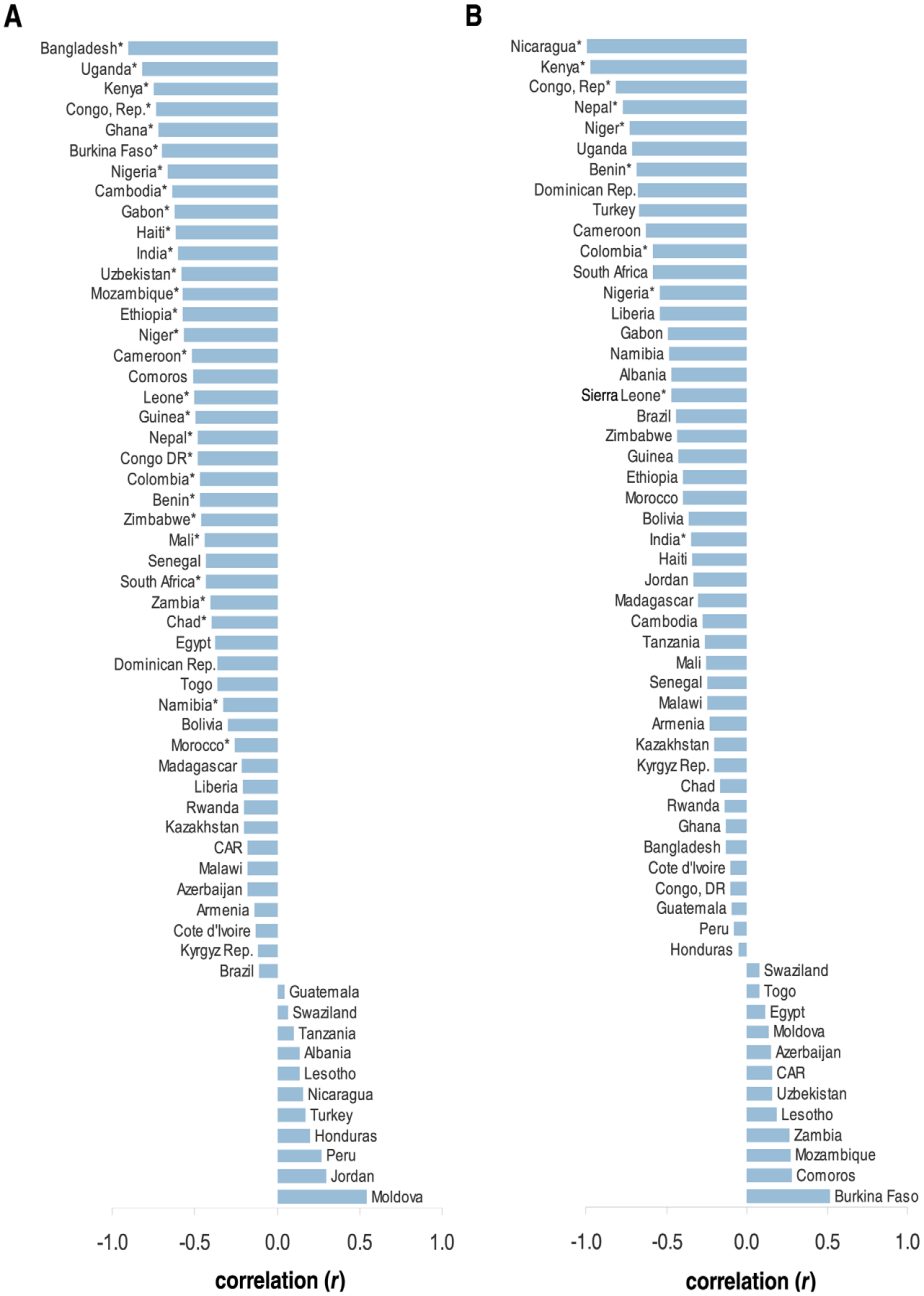
Within country (neighborhood-level) correlation of underweight and overweight among women aged 20–49 in 57 low- to middle-income countries, overall (A), and in low-socioeconomic status groups (B). Notes: Panel A adjusted for age, education, household wealth, and place of residence, Panel B adjusted for age and place of residence; *P<0.05.

Within-countries, age-adjusted models generally revealed an inverse correlation between underweight and overweight at the neighborhood-level, with statistically significant negative correlations observed that varied between −0.25 (*P* = 0.019) in Morocco to −0.90 (*P*<0.0001) in Bangladesh (**Figure 3A**). Overall, negative correlations between underweight and overweight were observed at the neighborhood-level in 46 of the 57 countries, and correlations were statistically significant (P<0.05) in 29 countries (see **Table S2** for significance levels). Although positive correlations in age-adjusted models were observed in 8 countries (Albania, Guatemala, Honduras, Lesotho, Nicaragua, Swaziland, Tanzania, Turkey), none were found to be statistically significant.

Globally, the results of analyses restricted to low-SES were largely similar to the full analysis; negative correlations in underweight and overweight were observed at the country (*r* = −0.79, *P*<0.001, **Figure 2B**) and neighborhood (*r* = −0.45, P<0.001) levels, with adjustment for age and place of residence (**Figure 3B**). Within countries, the inverse relationship was found to be statistically significant in 10/57 countries (see **Table S2** for significance levels). No countries were found to have a statistically significant positive correlation.

**Figure 4:**
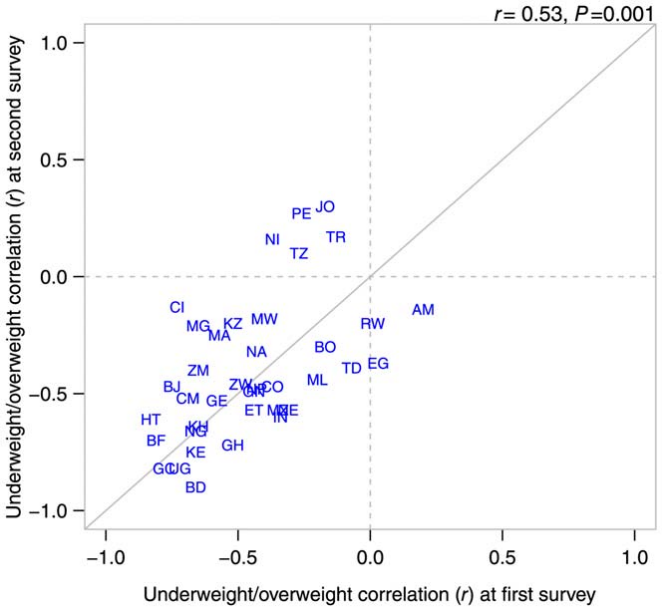
Within-country (neighborhood) and global (country and neighborhood level) age-adjusted correlation of underweight and overweight among women aged 20–49 at the first DHS survey (median year: 1996) *vs.* the most recent DHS survey (median year: 2005) in 36 low- to middle-income countries with repeated surveys. Notes: Models adjusted for age; **Country abbreviations**: AM Armenia, BD Bangladesh, BJ Benin, BO Bolivia, BF Burkina Faso, KH Cambodia, CM Cameroon, TD Chad, CO Colombia, CI Cote d'Ivoire, EG Egypt, ET Ethiopia, GH Ghana, GN Guinea, HT Haiti, IN India, JO Jordan, KZ Kazakhstan, KE Kenya, MG Madagascar, MW Malawi, ML Mali, MA Morocco, MZ Mozambique, NA Namibia, NP Nepal, NI Nicaragua, NE Niger, NG Nigeria, PE Peru, RW Rwanda, TZ Tanzania, TR Turkey, UG Uganda, ZM Zambia, ZW Zimbabwe, GC Global (country-level), GE Global (neighborhood-level).

In global age-adjusted analyses restricted to 36 countries with repeated surveys, correlations between underweight and overweight at the country- and neighborhood-levels were negative, statistically significant, nearly identical over time (median interval: 10 years), and to the full sample results (**Table S3**). The change over time within countries is represented in **Figure 4**, which plots the underweight/overweight correlation from the first versus the most recent survey for countries with repeated surveys.

Within the subset of countries with repeated surveys, 18/36 were found to have a negative and statistically correlation at the neighborhood-level in the first available survey (median year: 1996) (**Table S3**). In the most recent survey (median year: 2005), 23/36 countries demonstrated a statistically significant negative correlation (*P*<0.05). Among 19/36 countries, the correlation was found to be negative and statistically significant at both time points (**Table S3**). Five countries with negative correlations at the first survey period (Jordan, Nicaragua, Peru, Tanzania, and Turkey) were found to have positive correlations in the most recent survey. The correlation between underweight and overweight among these countries did not reach statistical significance at either survey point and consecutive positive correlations were not observed for any countries over time.

**Figure 5:**
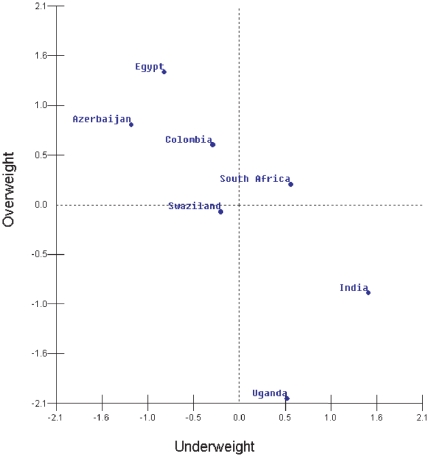
Scatter plot of country-level residuals for underweight and overweight among men aged 15–54 in 7 low- to middle-income countries (*r* = −0.72, *P* = 0.28).

We conducted several sensitivity analyses to explore the consistency of our findings. First, pooled and country-specific analyses were repeated on adult males (15–54 y) in a subset of 7 countries. The countries and sample sizes of males included in these analyses are presented in **Table 3**. The results of the pooled analysis for males showed an inverse relationship between underweight and overweight at the country-level similar to the corresponding analysis on females (**Figure 5**); although the finding for males was not statistically significant due to few countries. Within country analyses for males also demonstrated a similar pattern to the overall results, with negative and statistically significant correlations between underweight and overweight observed in 5/7 countries (P<0.05, **Table 3**) For Colombia and South Africa, data on all adult males (aged 15+) was analyzed.

**Table 3:** Global (country and neighborhood level) and within-country (neighborhood) age-adjusted correlation of underweight and overweight among men aged 15–54 from DHS surveys in seven low- to middle-income countries.

Country	*r*	*P*-value
**Global** *		
Country-level	−0.72	0.28
Neighborhood-level	−0.39	<0.0001
**Country (n)**		
Azerbaijan (2,484)	−0.23	0.36
Egypt (5,640)	−0.40	0.019
India (71,289)	−0.42	<0.0001
Swaziland (4,053)	−0.49	0.09
Uganda (2,456)	−0.65	0.002
Colombia (28,273)**	−0.18	0.025
South Africa (5,591)**	−0.56	<0.001

Notes:

*n = 7 countries; 10,746 neighborhoods; 119,786 individuals.

**(Age 15+ y).

Correlations are based on the neighborhood-level covariance in underweight and overweight from age-adjusted models.

Second, we restricted the pooled country-level analyses to women with only preschool aged children, some preschool aged children, or no children, and to women of younger (15–24) and middle (25–49) ages. The results of these analyses are presented in **Table 4**. In fully adjusted models for these subgroups, a consistently inverse and statistically significant association was observed between underweight and overweight was observed at the country level that varied between −0.70 and −0.78, compared to the correlation in the overall study sample of −0.78 (P<0.001). Finally, additional analyses were conducted on the South African (1998) and Colombian (2004) and surveys which included women older than 50 years of age. These results are presented in **Table 5**. In both countries, the correlations for the different age strata were consistent with the overall findings; however the South African analysis had less statistical precision due to sample size limitations. Negative and statistically significant correlations of large magnitude (*r*<−0.8) were observed among the older age groups in both countries, which may be reflective of older cohorts preceding the nutrition transition in these countries.

**Table 4:** Global (country level) age and fully adjusted correlation of underweight and overweight among women with and without children, and among women of younger and middle age groups in 57 low- to middle-income countries.

		Age adjusted		Fully adjusted**	
Case	N	Country-level *r*	*P*-value	Country-level *r*	*P*-value
Study sample*	451322	−0.79	0.000	−0.78	0.000
Women with all children 5 and younger	66042	−0.73	0.000	−0.70	0.000
Women with at least 1 child 5 and younger	250683	−0.77	0.000	−0.75	0.000
Women without children	48189	−0.72	0.000	−0.70	0.000
Women aged 12–24 y	210056	−0.72	0.000	−0.71	0.000
Women aged 25–49 y	353351	−0.80	0.000	−0.78	0.000

*Notes: Study sample includes all non-pregnant women aged 20–49 and included women with and without children of any age.

**Adjusted for age, education, household wealth, and place of residence.

**Table 5:** Within country correlations between underweight and overweight among women of younger, middle, and older age groups in Colombia and South Africa.

	Colombia			South Africa		
Case	N	*r* **	*P*-value	N	*r* **	*P*-value
Study sample*	27062	−0.55	0.000	4263	−0.54	0.010
Women aged 15–24 y	13063	−0.76	0.000	2038	−0.45	0.052
Women aged 25–49 y	23607	−0.71	0.000	3394	−0.46	0.086
Women aged 50+ years	7575	−0.80	0.018	2410	−0.88	0.002

**Notes:**

*Study sample includes all non-pregnant women aged 20–49 and included women with and without children of any age.

**Correlations adjusted for age, education, household wealth, and place of residence.

## Discussion

Using large representative samples of adult women of reproductive ages from 57 LMICs, we observed a robust negative correlation between underweight and overweight globally across countries and for neighborhoods within a majority of countries. This finding was consistent in analyses stratified by urban/rural location. The inverse correlation was mirrored among low SES groups globally and within countries, suggesting that underweight and overweight are not happening simultaneously in this group. Among the subset of countries with multiple measurements, the negative correlations observed at the country and neighborhood levels did not appear to change substantially in direction or magnitude over time.

Before we discuss these findings, we discuss the data limitations of the study. First, our analyses were restricted to women of reproductive age, due to our use of the Demographic and Health Surveys as the data source. Despite this limitation the DHS offers many advantages including comparable survey instruments, standardization of anthropometric measurements, sampling procedures and other methodology that facilitate pooling across surveys. Several studies investigating the coexistence of underweight and overweight have been based on reproductive-aged women, and this is largely due to the target population of the Demographic and Health Surveys (women aged 15–49) which provide an important source of nutrition-related data for LMICs [6], [37]–[40]. Whether the patterns observed in our study hold for women in other countries and of different ages and for adult men generally in comparable samples with objective measures of height and weight remains an open empirical question. The consistency of our findings among women of different age groups, with and without children, and among men (aged 15–54) in 7 countries during sensitivity analyses, however, suggests that our findings may be generalizable to other populations and settings. Second, the countries included in this study were not surveyed at the same time even though a majority of countries (45/57) were surveyed after 2000. Thirdly, it was beyond the scope of this study to investigate the double burden of disease more generally such as the coexistence of both infectious and noncommunicable diseases within a population [9]. The primary purpose of our study was to further previous research on the double burden of malnutrition, based largely on the prevalence of underweight and overweight, by advancing a methodology by which to objectively assess for the coexistence of underweight and overweight with a population. We focused specifically on the double burden of malnutrition within populations of adult women and, for a smaller group of countries, adult men in LMICs. Additionally, our focus on adults did not allow for the investigation of the coexistence underweight and overweight between parents and children or within the same household, although this has been highlighted as an issue of potential concern in some countries [41]–[42].

Our study findings, with objective height and weight measurements, suggest that the hypothesized double nutritional burden has yet to occur in a majority of LMICs. Among countries with repeated surveys, we observed that over time, the prevalence of overweight increased in nearly all countries, and while underweight decreased in two thirds of these countries, it was at a much slower rate. Such trends may have future implications especially for those countries were the prevalence of underweight remains substantial. Namibia, Sierra Leone, and South Africa appear to meet the criteria for a double nutritional burden with the coexistence of above average levels of underweight and overweight. However, for all three countries a statistically significant negative correlation between underweight and overweight was observed at the level of neighborhoods within the countries, suggesting that the prevalence of underweight and overweight within the population may be geographically patterned. Similar results have been reported in within-country studies from Bangladesh and India using the same methodology [43]–[44]. Further, to the extent that the prevalence of obesity is increasing in LMICs, it has been shown that within countries, overweight is concentrated primarily among the high socioeconomic groups [39]–[40], [45].

Our study highlighted several countries, notably Jordan, Peru, and Turkey, in which a shift from a negative to positive correlation in underweight and overweight was observed over median period of 10 years. Kazakhstan also demonstrated a large change in the magnitude of the correlation but remained negative in both surveys. These countries are among 14 countries in our sample considered upper-middle income with a per capita Gross Domestic Product (pcGDP) of ≥$3946 [46]. While plausible, the implication that a coexistence of underweight and overweight emerges at higher levels of economic development was not statistically supported in this study. The few upper-middle income countries that demonstrated increases in the underweight/overweight correlation appear to be exceptions to the trend and such findings may not be generalizable to the lower-middle and low-income countries. Among the remaining countries, no discernable relationship was found between change in neighborhood-level underweight/overweight correlation and pcGDP. Interestingly, other upper-middle income countries such as Colombia and Namibia demonstrated strong inverse and statistically significant correlations in underweight and overweight further suggesting that the double burden of malnutrition may be related to income-inequality within countries, rather than overall economic development [47].

Although many low and middle income countries face problems of underweight and overweight, the hypothesized “double burden” of malnutrition has not definitively occurred either among adult women of reproductive age in a majority of the LMICs studied or among adult males studied in a smaller group of countries. While the double burden of nutrition may indeed be forthcoming within the context of epidemiologic transition, such a characterization seems inappropriate at present for most LMICs in light of our findings of an explicitly inverse association between the prevalence of underweight and overweight at the level of countries and small geographic areas and, even more importantly, that underweight and overweight are unequivocally segregated into two separate socioeconomic groups [39], [44]. The scientific and policy narratives related to the double burden of malnutrition in LMICs need to be evidence-based in order to be focused and fair.

## Supporting Information

Table S1 **Year, sampling plan, and response rates for demographic and health (DHS) surveys conducted in 57 low- and middle-income countries.**
(DOC)

Table S2 **Within country (neighborhood-level) correlation and significance level of coexistence of underweight and overweight among women aged 20–49 in 57 low- to middle-income countries, by overall, urban, rural, and low-socioeconomic status samples.**
(DOC)

Table S3 **Global (country and neighborhood-level) and within-country (neighborhood) age-adjusted correlation of underweight and overweight among women aged 20–49 from 36 low- to middle-income countries with repeated surveys.**
(DOC)
