# Supplementary material for: Global Burden of Double Malnutrition: Has Anyone Seen It?
Source: PLoS One. 2011 Sep 28;6(9):e25120. doi: 10.1371/journal.pone.0025120 (PMC3182195; doi:10.1371/journal.pone.0025120)
Supplement: Table S1 — Year, sampling plan, and response rates for demographic and health (DHS) surveys conducted in 57 low- and middle-income countries. (DOC) [file pone.0025120.s001.doc]

**Table S1** Year, sampling plan, and response rates for demographic and health (DHS) surveys conducted in 57 low- and middle-income countries.

| **Country** | **Survey Year** | **Sampling plan** | **Overall**  **response rate** |
| --- | --- | --- | --- |
| Albania | 2008 | 2-stage cluster, not self-weighting | 96.0 |
| Armenia | 2005 | 2-stage cluster | 92.8 |
|  | 2000 | 2-stage cluster | 93.5 |
| Azerbaijan | 2006 | 2-stage cluster, not self-weighting, autonomous Republic of Nakhichevan excluded | 95.5 |
| Bangladesh* | 2007 | 2-stage cluster | 97.8 |
|  | 1996 | 2-stage cluster, not self-weighting, 3 clusters excluded due to remoteness | 96.9 |
| Benin | 2006 | 2-stage cluster, self-weighting within strata | 93.5 |
|  | 1996 | 2-stage cluster, self-weighting within strata | 94.7 |
| Bolivia | 2003 | 2-stage cluster, not self-weighting | 94.4 |
|  | 1994 | 2-stage cluster | 92.3 |
| Brazil^a^ | 1996 | 2-stage cluster, rural Northern area in Tocantins excluded | 75.1 |
| Burkina Faso | 2003 | 2-stage cluster, not self-weighting | 95.7 |
|  | 1992 | 2-stage cluster, self-weighting within urban/rural/capital strata | 87.6 |
| Cambodia^b^ | 2005 | 2-stage cluster, not self-weighting | 91.2 |
|  | 2000 | 3-stage cluster | 97.9 |
| Cameroon^b^ | 2005 | 2-stage cluster, not self-weighting, 1 selected cluster not covered due to remoteness | 92.0 |
|  | 1998 | 2-stage cluster, self-weighting within strata | 93.6 |
| Central African Republic (CAR)^c^ | 1995 | 2-stage cluster, self-weighting within strata | 97.4 |
| Chad | 2004 | 2-stage cluster, self-weighting within strata, Ouaddaï prefecture excluded | 94.7 |
|  | 1996 | 2-stage cluster, self-weighting within strata |  |
| Colombia | 2005 | 2-stage cluster, self-weighting within strata | 81.7 |
|  | 1995 | 2-stage cluster, not self-weighting, former national territories excluded | 92.2 |
| Comoros^c^ | 1996 | 2-stage cluster | 95.4 |
| Congo, Dem. Rep.^b^ | 2007 | 2-stage urban, 3-stage rural cluster | 96.0 |
| Congo, Rep. | 2005 | 2-stage cluster | 94.0 |
| Cote d'Ivoire | 1998-9 | 2-stage cluster, self-weighting within strata | 94.4 |
|  | 1994 | 3-stage urban, 2-stage rural cluster, self-weighting within strata | 95.1 |
| Dominican Republic | 1996 | 2-stage cluster, self-weighting within health region strata | 91.1 |
| Egypt* | 2008 | 3-stage cluster, not self-weighting | 98.8 |
|  | 1995 | 3-stage cluster, not self-weighting | 98.6 |
| Ethiopia^b^ | 2005 | 2-stage cluster, not self-weighting, 2 regions with nomadic populations excluded | 94.2 |
|  | 2000 | 2-stage cluster | 97.1 |
| Gabon | 2000 | 2-stage cluster, not self-weighting | 91.4 |
| Ghana | 2008 | 2-stage cluster | 95.1 |
|  | 1993 | 2-stage cluster | 97.1 |
| Guatemala | 1998-9 | 2-stage cluster | 78.1 |
| Guinea | 2005 | 2-stage cluster | 96.4 |
|  | 1999 | 2-stage cluster, self-weighting within strata | 92.6 |
| Haiti | 2005-6 | 2-stage cluster, not self-weighting | 98.4 |
|  | 1994 | 2-stage cluster, self-weighting within strata | 91.4 |
| Honduras | 2005 | 2-stage cluster | 90.3 |
| India | 2005 | 3-stage urban, 2-stage rural cluster, self-weighting within urban/rural strata | 92.3 |
|  | 1998 | 3-stage urban, 2-stage rural cluster, self-weighting, Union territories excluded | 95.5 |
| Jordan*^b^ | 2007 | 2-stage cluster, not self-weighting | 96.7 |
|  | 1997 | 2-stage cluster, self-weighting within each governate | 93.0 |
| Kazakhstan^b^ | 1999 | 2-stage cluster, not self-weighting | 95.9 |
|  | 1995 | 2-stage cluster, self-weighting within strata | 96.7 |
| Kenya | 2003 | 2-stage cluster, not self-weighting | 90.5 |
|  | 1998 | 2-stage cluster, not self-weighting | 92.6 |
| Kyrgyz Republic | 1997 | 3-stage cluster, not self-weighting | 96.7 |
| Lesotho^b^ | 2004 | 2-stage cluster | 89.8 |
| Liberia | 2007 | 2-stage cluster, not self-weighting | 92.5 |
| Madagascar | 2003-4 | 2-stage cluster | 93.1 |
|  | 1997 | 2-stage cluster, self-weighting within strata | 92.8 |
| Malawi | 2004 | 2-stage cluster, not self-weighting | 93.6 |
|  | 1992 | 2-stage cluster, self-weighting within strata | 95.4 |
| Mali | 2006 | 2-stage cluster | 95.4 |
|  | 1995 | 2-stage cluster, self-weighting within strata | 94.8 |
| Moldova | 2005 | 2-stage cluster, not self-weighting | 93.6 |
| Morocco | 2003-4 | 2-stage cluster, not self-weighting | 95.1 |
|  | 1992 | 2-stage cluster, self-weighting within strata | 95.7 |
| Mozambique | 2003 | 2-stage cluster | 86.2 |
|  | 1997 | 2-stage cluster, not self-weighting | 87.8 |
| Namibia | 2006-7 | 2-stage cluster | 92.6 |
|  | 1992 | 2-stage cluster, self-weighting within strata | 84.3 |
| Nepal | 2006 | 2-stage cluster, not self-weighting | 98.0 |
|  | 1996 | 2-stage cluster, self-weighting at urban/rural areas within strata | 98.2 |
| Nicaragua | 2001 | 2-stage cluster, not self-weighting | 87.4 |
|  | 1997 | 2-stage cluster, self-weighting within strata | 87.7 |
| Niger^b^ | 2006 | 2-stage cluster, not self-weighting | 93.6 |
|  | 1998 | 2-stage cluster, self-weighting within strata | 95.1 |
| Nigeria | 2008 | 2-stage cluster | 94.9 |
|  | 2003 | 2-stage cluster | 94.1 |
| Peru^b^ | 2004 | 3-stage cluster, not self-weighting | 94.9 |
|  | 1991 | 2-stage urban, 3-stage rural cluster | 96.9 |
| Rwanda^b^ | 2005 | 2-stage cluster, not self-weighting | 97.9 |
|  | 2000 | 2-stage cluster, not self-weighting | 97.6 |
| Senegal^d^ | 2005 | 2-stage cluster | 92.3 |
| Sierra Leone | 2008 | 2-stage cluster | 91.8 |
| South Africa | 1998 | 2-stage cluster, self-weighting within strata | 92.3 |
| Swaziland | 2006 | 2-stage cluster | 89.2 |
| Tanzania | 2004-5 | 2-stage cluster, not self-weighting | 96.1 |
|  | 1996 | 3-stage cluster, self-weighting at urban/rural areas within strata | 93.5 |
| Togo^c^ | 1998 | 2-stage cluster, self-weighting within strata | 94.3 |
| Turkey* | 1998 | 2-stage cluster, self-weighting within strata | 84.9 |
|  | 1993 | 2-stage cluster, not self-weighting | 92.0 |
| Uganda^d^ | 2006 | 2-stage cluster, not self-weighting | 92.3 |
|  | 1995 | 2-stage cluster, not self-weighting | 94.3 |
| Uzbekistan | 1996 | 2-stage cluster, not self-weighting | 95.6 |
| Zambia | 2007 | 2-stage cluster | 94.4 |
|  | 1996 | 3-stage cluster, self-weighting within strata | 95.6 |
| Zimbabwe | 2005-6 | 2-stage cluster, not self-weighting | 85.6 |
|  | 1994 | 2-stage cluster, not self-weighting | 94.2 |

Notes:

*Ever-married sample

^A^ Height & weight measured for women with births in 5 years prior to survey

^B^ Height & weight measured in ½ subsample of women

^C^ Height & weight measured for women with births in 3 years prior to survey

^D^ Height & weight measured in 1/3 subsample of women

See [www.measuredhs.com/pubs/search/search_results.cfm?Type=5&srchTp=type&newSrch=1](http://www.measuredhs.com/pubs/search/search_results.cfm?Type=5&srchTp=type&newSrch=1) for final survey reports by country
