# Supplementary material for: Global Burden of Double Malnutrition: Has Anyone Seen It?
Source: PLoS One. 2011 Sep 28;6(9):e25120. doi: 10.1371/journal.pone.0025120 (PMC3182195; doi:10.1371/journal.pone.0025120)
Supplement: Table S2 — Within country (neighborhood-level) correlation and significance level of coexistence of underweight and overweight among women aged 20–49 in 57 low- to middle-income countries, by overall, urban, rural, and low-socioeconomic status samples. (DOC) [file pone.0025120.s002.doc]

**Table S2** Within country (neighborhood-level) correlation and significance level of coexistence of underweight and overweight among women aged 20-49 y in 57 low- to middle-income countries, by overall, urban, rural, and low-socioeconomic status samples

| **Country** | **All** | | **Urban** | | **Rural** | | **Low SES** | |
| --- | --- | --- | --- | --- | --- | --- | --- | --- |
| Underweight/Overweight | Age-adjusted | | Age-adjusted | | Age-adjusted | | Age and location adjusted | |
|  | *r* | *P-value* | *r* | *P-value* | *r* | *P-value* | *r* | *P-value* |
| Albania | 0.14 | 0.565 | -0.07 | 0.811 | 0.27 | 0.569 | -0.47 | 0.201 |
| Armenia | -0.14 | 0.623 | -0.05 | 0.865 | -0.25 | 0.638 | -0.23 | 0.648 |
| Azerbaijan | -0.18 | 0.350 | -0.56 | 0.124 | -0.28 | 0.269 | 0.15 | 0.644 |
| Bangladesh | -0.90 | 0.000 | -0.87 | 0.000 | -0.85 | 0.000 | -0.13 | 0.818 |
| Benin | -0.47 | 0.000 | -0.64 | 0.000 | -0.47 | 0.000 | -0.68 | 0.000 |
| Bolivia | -0.30 | 0.218 | -0.05 | 0.906 | -0.37 | 0.127 | -0.36 | 0.187 |
| Brazil | -0.11 | 0.742 | -0.47 | 0.274 | -0.60 | 0.200 | -0.44 | 0.266 |
| Burkina Faso | -0.70 | 0.000 | -0.56 | 0.022 | -0.56 | 0.000 | 0.52 | 0.248 |
| Cambodia | -0.64 | 0.000 | -0.62 | 0.037 | -0.43 | 0.063 | -0.27 | 0.649 |
| Cameroon | -0.52 | 0.001 | -0.66 | 0.001 | -0.55 | 0.013 | -0.63 | 0.053 |
| Central African Republic | -0.18 | 0.593 | 0.06 | 0.920 | -0.20 | 0.811 | 0.16 | 0.815 |
| Chad | -0.39 | 0.011 | -0.32 | 0.370 | -0.05 | 0.888 | -0.16 | 0.773 |
| Colombia | -0.47 | 0.000 | -0.53 | 0.000 | -0.61 | 0.002 | -0.58 | 0.003 |
| Comoros | -0.51 | 0.206 | 0.31 | 0.847 | -0.48 | 0.302 | 0.28 | 0.820 |
| Congo, Dem. Rep. | -0.48 | 0.003 | -0.78 | 0.004 | -0.61 | 0.006 | -0.10 | 0.793 |
| Congo, Rep. | -0.73 | 0.000 | -0.88 | 0.000 | -0.87 | 0.000 | -0.81 | 0.001 |
| Cote d'Ivoire | -0.13 | 0.643 | -0.18 | 0.640 | -0.10 | 0.867 | -0.10 | 0.872 |
| Dominican Republic | -0.36 | 0.085 | -0.14 | 0.753 | -0.45 | 0.094 | -0.67 | 0.097 |
| Egypt | -0.37 | 0.141 | -0.67 | 0.013 | -0.12 | 0.682 | 0.12 | 0.820 |
| Ethiopia | -0.57 | 0.000 | -0.75 | 0.019 | -0.33 | 0.256 | -0.40 | 0.262 |
| Gabon | -0.62 | 0.006 | -0.46 | 0.512 | -0.72 | 0.028 | -0.49 | 0.212 |
| Ghana | -0.72 | 0.000 | -0.47 | 0.167 | -0.52 | 0.072 | -0.13 | 0.779 |
| Guatemala | 0.05 | 0.813 | -0.86 | 0.044 | -0.02 | 0.950 | -0.10 | 0.816 |
| Guinea | -0.49 | 0.006 | -0.91 | 0.022 | -0.64 | 0.017 | -0.43 | 0.477 |
| Haiti | -0.61 | 0.000 | -0.76 | 0.002 | -0.47 | 0.070 | -0.34 | 0.575 |
| Honduras | 0.20 | 0.264 | -0.24 | 0.455 | 0.23 | 0.250 | -0.05 | 0.867 |
| India | -0.60 | 0.000 | -0.44 | 0.000 | -0.45 | 0.000 | -0.34 | 0.000 |
| Jordan | 0.30 | 0.632 | -0.01 | 0.992 | -0.71 | 0.083 | -0.33 | 0.516 |
| Kazakhstan | -0.20 | 0.640 | -0.14 | 0.793 | -0.32 | 0.632 | -0.21 | 0.686 |
| Kenya | -0.75 | 0.000 | -0.83 | 0.000 | -0.72 | 0.000 | -0.97 | 0.000 |
| Kyrgyz Republic | -0.12 | 0.808 | 0.22 | 0.769 | -0.25 | 0.611 | -0.20 | 0.660 |
| Lesotho | 0.14 | 0.793 | -0.69 | 0.431 | 0.56 | 0.162 | 0.19 | 0.787 |
| Liberia | -0.21 | 0.254 | -0.30 | 0.349 | -0.19 | 0.511 | -0.54 | 0.085 |
| Madagascar | -0.21 | 0.061 | -0.26 | 0.336 | -0.25 | 0.110 | -0.31 | 0.377 |
| Malawi | -0.18 | 0.289 | -0.30 | 0.667 | -0.07 | 0.767 | -0.24 | 0.662 |
| Mali | -0.44 | 0.000 | -0.64 | 0.011 | -0.52 | 0.001 | -0.25 | 0.478 |
| Moldova | 0.55 | 0.246 | 0.16 | 0.751 | 0.20 | 0.641 | 0.14 | 0.800 |
| Morocco | -0.25 | 0.019 | 0.19 | 0.397 | -0.55 | 0.001 | -0.40 | 0.051 |
| Mozambique | -0.57 | 0.000 | -0.69 | 0.001 | -0.37 | 0.051 | 0.28 | 0.539 |
| Namibia | -0.32 | 0.013 | -0.13 | 0.586 | -0.06 | 0.799 | -0.49 | 0.189 |
| Nepal | -0.48 | 0.000 | -0.79 | 0.000 | -0.40 | 0.001 | -0.77 | 0.004 |
| Nicaragua | 0.16 | 0.462 | -0.09 | 0.488 | -0.11 | 0.779 | -0.99 | 0.000 |
| Niger | -0.57 | 0.000 | -0.53 | 0.075 | -0.66 | 0.001 | -0.73 | 0.018 |
| Nigeria | -0.66 | 0.000 | -0.57 | 0.000 | -0.68 | 0.000 | -0.54 | 0.000 |
| Peru | 0.27 | 0.236 | -0.01 | 0.975 | 0.13 | 0.643 | -0.08 | 0.799 |
| Rwanda | -0.20 | 0.541 | -0.60 | 0.102 | -0.49 | 0.188 | -0.14 | 0.784 |
| Senegal | -0.43 | 0.051 | -0.53 | 0.084 | -0.43 | 0.269 | -0.25 | 0.592 |
| Sierra Leone | -0.50 | 0.000 | -0.49 | 0.038 | -0.50 | 0.002 | -0.47 | 0.042 |
| South Africa | -0.43 | 0.030 | -0.33 | 0.215 | -0.75 | 0.007 | -0.58 | 0.139 |
| Swaziland | 0.07 | 0.818 | -0.12 | 0.882 | -0.12 | 0.765 | 0.08 | 0.906 |
| Tanzania | 0.10 | 0.343 | -0.23 | 0.442 | 0.19 | 0.144 | -0.26 | 0.429 |
| Togo | -0.36 | 0.109 | -0.34 | 0.645 | -0.23 | 0.395 | 0.08 | 0.820 |
| Turkey | 0.17 | 0.678 | 0.27 | 0.507 | -0.40 | 0.357 | -0.67 | 0.196 |
| Uganda | -0.82 | 0.000 | -0.15 | 0.836 | -0.75 | 0.000 | -0.71 | 0.067 |
| Uzbekistan | -0.58 | 0.004 | -0.79 | 0.001 | -0.51 | 0.190 | 0.16 | 0.794 |
| Zambia | -0.40 | 0.020 | -0.77 | 0.033 | -0.50 | 0.022 | 0.27 | 0.581 |
| Zimbabwe | -0.46 | 0.005 | -0.72 | 0.896 | -0.64 | 0.002 | -0.43 | 0.167 |

Notes: Correlations are based on the neighborhood-level covariance in underweight and overweight from age-adjusted models, except the low SES sample which is also adjusted for urban/rural location (place of residence). Significance tests are based on two-tailed Wald tests using chi square distribution.
