# Supplementary material for: Global Burden of Double Malnutrition: Has Anyone Seen It?
Source: PLoS One. 2011 Sep 28;6(9):e25120. doi: 10.1371/journal.pone.0025120 (PMC3182195; doi:10.1371/journal.pone.0025120)
Supplement: Table S3 — Global (country and neighborhood-level) and within-country (neighborhood) age-adjusted correlation of underweight and overweight among women aged 20–49 from 36 low- to middle-income countries with repeated surveys. (DOC) [file pone.0025120.s003.doc]

**Table S3** Global (country and neighborhood-level) and within-country (neighborhood) age-adjusted correlation of underweight and overweight among women aged 20-49 y from 36 low- to middle-income countries with repeated surveys*

|  | **First survey** | | | **Most recent survey** | | |
| --- | --- | --- | --- | --- | --- | --- |
| **Country** | Year | *r* | *P-value* | Year | *r* | *p-value* |
| **Global** |  |  |  |  |  |  |
| Country-level | 1996 | -0.78 | 0.001 | 2005 | -0.82 | 0.001 |
| Neighborhood-level | 1996 | -0.58 | 0.000 | 2005 | -0.53 | 0.000 |
| **Country** |  |  |  |  |  |  |
| Armenia | 2000 | 0.20 | 0.542 | 2005 | -0.14 | 0.623 |
| Bangladesh | 1996 | -0.66 | 0.000 | 2007 | -0.90 | 0.000 |
| Benin | 1996 | -0.75 | 0.001 | 2006 | -0.47 | 0.000 |
| Bolivia | 1993 | -0.17 | 0.677 | 2003 | -0.30 | 0.218 |
| Burkina Faso | 1992 | -0.81 | 0.001 | 2003 | -0.70 | 0.000 |
| Cambodia | 2000 | -0.65 | 0.001 | 2005 | -0.64 | 0.000 |
| Cameroon | 1998 | -0.69 | 0.001 | 2005 | -0.52 | 0.001 |
| Chad | 1996 | -0.07 | 0.592 | 2004 | -0.39 | 0.011 |
| Colombia | 1995 | -0.37 | 0.416 | 2005 | -0.47 | 0.000 |
| Cote d'Ivoire | 1994 | -0.73 | 0.001 | 2005 | -0.13 | 0.643 |
| Egypt | 1995 | 0.03 | 0.906 | 2008 | -0.37 | 0.141 |
| Ethiopia | 2000 | -0.44 | 0.000 | 2005 | -0.57 | 0.000 |
| Ghana | 1993 | -0.52 | 0.093 | 2008 | -0.72 | 0.000 |
| Guinea | 1999 | -0.44 | 0.046 | 2005 | -0.49 | 0.006 |
| Haiti | 1994 | -0.83 | 0.002 | 2005 | -0.61 | 0.000 |
| India | 1998 | -0.34 | 0.001 | 2005 | -0.60 | 0.000 |
| Jordan | 1997 | -0.17 | 0.537 | 2007 | 0.30 | 0.632 |
| Kazakhstan | 1995 | -0.52 | 0.052 | 1999 | -0.20 | 0.640 |
| Kenya | 1998 | -0.66 | 0.005 | 2003 | -0.75 | 0.000 |
| Madagascar | 1997 | -0.65 | 0.014 | 2003 | -0.21 | 0.061 |
| Malawi | 1992 | -0.40 | 0.121 | 2004 | -0.18 | 0.289 |
| Mali | 1995 | -0.20 | 0.276 | 2006 | -0.44 | 0.000 |
| Morocco | 1992 | -0.57 | 0.010 | 2003 | -0.25 | 0.019 |
| Mozambique | 1997 | -0.35 | 0.067 | 2003 | -0.57 | 0.000 |
| Namibia | 1992 | -0.43 | 0.042 | 2006 | -0.32 | 0.013 |
| Nepal | 1996 | -0.43 | 0.042 | 2006 | -0.48 | 0.000 |
| Nicaragua | 1997 | -0.37 | 0.123 | 2001 | 0.16 | 0.462 |
| Niger | 1998 | -0.31 | 0.109 | 2006 | -0.57 | 0.000 |
| Nigeria | 2003 | -0.66 | 0.000 | 2008 | -0.66 | 0.000 |
| Peru | 1991 | -0.26 | 0.626 | 2004 | 0.27 | 0.236 |
| Rwanda | 2000 | 0.01 | 0.944 | 2005 | -0.20 | 0.541 |
| Tanzania | 1996 | -0.27 | 0.174 | 2004 | 0.10 | 0.343 |
| Turkey | 1993 | -0.13 | 0.738 | 1998 | 0.17 | 0.678 |
| Uganda | 1995 | -0.72 | 0.000 | 2006 | -0.82 | 0.000 |
| Zambia | 1996 | -0.65 | 0.003 | 2007 | -0.40 | 0.020 |
| Zimbabwe | 1994 | -0.49 | 0.095 | 2005 | -0.46 | 0.005 |

Notes: *Median year of first survey was 1996 and most recent survey was 2005

Correlations based on the neighborhood-level covariance in underweight and overweight from age-adjusted models, significance tests based on two-tailed Wald tests using chi square distribution.
